# Using the MitoB method to assess levels of reactive oxygen species in ecological studies of oxidative stress

**DOI:** 10.1038/srep41228

**Published:** 2017-01-24

**Authors:** Karine Salin, Sonya K. Auer, Eugenia M. Villasevil, Graeme J. Anderson, Andrew G. Cairns, William Mullen, Richard C. Hartley, Neil B. Metcalfe

**Affiliations:** 1 Institute of Biodiversity, Animal Health & Comparative Medicine, University of Glasgow, UK; 2 School of Chemistry, University of Glasgow, UK; 3 Institute of Cardiovascular and Medical Sciences, University of Glasgow, UK

## Abstract

In recent years evolutionary ecologists have become increasingly interested in the effects of reactive oxygen species (ROS) on the life-histories of animals. ROS levels have mostly been inferred indirectly due to the limitations of estimating ROS from *in vitro* methods. However, measuring ROS (hydrogen peroxide, H_2_O_2_) content *in vivo* is now possible using the MitoB probe. Here, we extend and refine the MitoB method to make it suitable for ecological studies of oxidative stress using the brown trout *Salmo trutta* as model. The MitoB method allows an evaluation of H_2_O_2_ levels in living organisms over a timescale from hours to days. The method is flexible with regard to the duration of exposure and initial concentration of the MitoB probe, and there is no transfer of the MitoB probe between fish. H_2_O_2_ levels were consistent across subsamples of the same liver but differed between muscle subsamples and between tissues of the same animal. The MitoB method provides a convenient method for measuring ROS levels in living animals over a significant period of time. Given its wide range of possible applications, it opens the opportunity to study the role of ROS in mediating life history trade-offs in ecological settings.

Over the last two decades evolutionary ecologists have shown a growing interest in the role of oxidative stress in shaping life-histories^1^,^2^,^3^,^4^. Oxidative stress occurs when the generation of reactive oxygen species (ROS) in an organism exceeds the capacity of its antioxidant defence and repair mechanisms to combat its effects, thereby causing the accumulation of oxidative damage^5^. Ecological studies investigating the effect of oxidative stress on life-histories have typically involved measurements of oxidative damage and/or the antioxidant system^6^,^7^,^8^,^9^,^10^,^11^,^12^. However, measurements of ROS have barely been attempted due to the complexity and specialized nature of the available methods.

The vast majority of the ROS present in organisms are generated by the mitochondria^13^, and have traditionally been measured through *in vitro* assays of the rate of mitochondrial ROS production^14^. However, these assays measure mitochondrial ROS generated under highly artificial levels of oxygen and substrate availability^15^,^16^,^17^. Thus, there are significant limitations to extrapolating *in vitro* results to the *in vivo* situation^18^. In addition, *in vitro* assays require considerable expertise and laboratory facilities and must be conducted on fresh samples (to ensure that the mitochondria are functional), so virtually precluding assays of ROS in wild animals.

Recently, Cochemé, *et al*.^19^ described an assay that measures ROS in living animals and offers the possibility to store samples prior to analysis, so finally overcoming the main limitations of *in vitro* assays. This method uses a newly-developed ratiometric probe, called MitoB, to measure levels of one major ROS, hydrogen peroxide (H_2_O_2_), within living animals^19^. When MitoB is administered to the living organism, it becomes concentrated in the mitochondria where it is converted by H_2_O_2_ into MitoP (Fig. 1). The selectivity of MitoB for mitochondrial H_2_O_2_ is based on the fact that it becomes almost entirely localized in the mitochondria because it is a lipophilic cation, and there it undergoes a specific reaction with H_2_O_2_ to give MitoP^19^, which chemically cannot be produced by other biological molecules (except peroxynitrite, which would reach mitochondria only under particular pathological circumstances^20^). Tissue samples from the animal can then be flash frozen for subsequent extraction and quantification of the compounds MitoB and MitoP. The mitochondrial H_2_O_2_ level is then related to the proportion of MitoB that has been converted into MitoP, expressed as the MitoP/MitoB ratio (Fig. 1). A high MitoP/MitoB ratio indicates that the mitochondria have a high average level of H_2_O_2_ over the period of MitoB exposure; the ratio thus provides an estimate of the imbalance between the generation and scavenging of H_2_O_2_ in the mitochondria. Importantly, the conversion of MitoB to MitoP by H_2_O_2_ is about ten million times slower than the catabolism of H_2_O_2_ by the main mitochondrial peroxidase, so that MitoB does not alter physiological levels of H_2_O_2_^19^.

To date, this method has only been employed in a biomedical context using cultured cells and model organisms (fly *Drosophila*, worm *Caenorhabditis elegans* and laboratory mouse *Mus musculus*^18^,^19^,^21^,^22^), with the sole exception of our recent study using a species of fish, the brown trout *Salmo trutta*^23^. The MitoB method appears to be applicable to various animal models. However, several of the steps in the MitoB protocol need to be validated before it can be used in a broader range of species and contexts (in particular, its potential for use in the natural population).

The aim of this article is to provide a detailed description of how to develop the MitoB protocol in organisms for which the method has never been applied before, with a general focus on issues that are likely to face ecologists and evolutionary biologists working on oxidative stress. We illustrate this by demonstrating how the protocols established for *Drosophila* by Cochemé, *et al*.^21^ had to be validated and extended for a new model, in our case brown trout^23^. Our previous paper showed that the MitoB method can be used in fish to study the relationship between mitochondrial ROS and metabolic rate^23^. Now, we explain in detail how the method was optimized for this new species, and also provide guidance on adapting this useful approach to other organisms in ecological studies. The steps needed to develop the MitoB method in a new organism were to (1) Establish the kinetics of the MitoB and MitoP compounds; (2) Verify whether the MitoP/MitoB ratio is consistent within and among tissues of an individual; (3) Determine whether exposure duration and initial concentration of MitoB affect MitoP/MitoB ratios; (4) Test whether the MitoB and MitoP molecules can transfer between fish and water, and so potentially between fish living in the same water body. By doing so, we establish that the method is suitable for aquatic organisms and for use in ecological studies of oxidative stress.

## Experiments and Results

### Step 1: Measurement of the time-course of MitoB and MitoP compounds in the animal

MitoB is slowly converted into MitoP, while MitoB and MitoP compounds are progressively excreted by the animal^21^, but the rates of conversion and excretion may differ between animal models. Thus, an important step is to measure the time-course of the MitoB and MitoP compounds in each new organism model to determine the appropriate exposure duration of MitoB: a sufficient time has elapsed when detectable amounts of MitoP have accumulated yet sufficient MitoB still remains in the animal^21^. Moreover, it is necessary to confirm that the rate of disappearance is similar between MitoB and MitoP so that the ratio of these compounds is not altered by differential kinetics of the two molecules. To assess the time course of the two compounds in trout, some fish received an injection of MitoP (50 nmol/fish, n = 20 fish) while others were injected with MitoB (50 nmol/fish, n = 20 fish). The rate of disappearance of MitoB and MitoP in the fish was assessed by recording the amount of MitoB or MitoP still present in a liver sample 3 h, 12 h, 24 h, 48 h and 72 h post-injection (n = 4 fish per time point). Statistical procedures were performed using IBM SPSS Statistics 22 (SPSS Inc., Chicago, IL, USA).

There was no detectable difference between MitoB and MitoP in their rate of disappearance from the liver (MitoB = −15.21 ± 9.87 pmol h^−1^; MitoP = −6.51 ± 9.80 pmol h^−1^; F_1, 34_ = 0.391, *P* = 0.536) after controlling for effects of the mass of liver used for the extraction of Mito compounds (F_1, 34_ = 0.238, *P* = 0.023). These results suggest that the MitoP/MitoB ratio in trout is unlikely to be affected by differential rates of disappearance of the two molecules, at least over the 72 h period covered by this trial. Moreover, the probe appears to accumulate rapidly within the tissues, as indicated by the large amount of MitoB present in the liver within 3 hours of injection (Table S3). MitoP was already detectable in some individuals 3 hours after injection with MitoB. However, in some cases MitoP was detected by the HPLC-MS but levels were still too low after 24 h to be accurately quantified (i.e. below the reliable detection limit of the HPLC-MS; Table S3). Finally, our results reveal that MitoB was still at high levels in the fish (i.e. had not all been excreted or converted to MitoP) 72 h after injection (Table S3). The sample size in the time course of MitoB experiment (n = 4 per time point) did not allow us to test conclusively as to whether the time-normalized MitoP/MitoB ratio remained constant over time. Thus, the exposure duration was set just above 24 h (range from 26 h to 29 h – see step 3 for discussion on effect of precise exposure duration on MitoP/MitoB ratio) for the rest of the study.

### Step 2: Determination of within- and between-tissue variation in MitoB and MitoP content and MitoP/MitoB ratio

In order to evaluate whether the H_2_O_2_ level, estimated as the MitoP/MitoB ratio, is consistent within a tissue and within an organism, liver and white muscle were sampled simultaneously from 20 fish exposed to MitoB for 26 h (26.15 ± 0.02 h), with two aliquots from each tissue being flash-frozen for separate extraction of compounds. White muscle was taken dorsally to the lateral line (to avoid contamination with red fibres) and just behind the caudal fin; one aliquot was collected from one side of the fish while the other aliquot was collected from the other side. Intra-class correlation coefficients (ICC) were used to test for the consistency of the quantification of the compounds between the two measures from the same tissue extract. The consistency was calculated using a two-way mixed effect ICC with an absolute agreement definition of concordance. This type of ICC was employed for all ICC used in our study as it assesses not only the correlation but also the exact similitude between measurements.

The consistency of the MitoP/MitoB ratio between two subsamples from the same individual was high for the liver (ICC *r* = 0.785, *P* = 0.001), but not for the white muscle (ICC *r* = 0.007, *P* = 0.486). White muscle showed a significantly higher average MitoP/MitoB ratio than the liver within individuals (0.266 ± 0.028 and 0.073 ± 0.008 respectively, paired t test: *t* = 6.711, df = 19, *P* < 0.001). However, there was no correlation between the individual MitoP/MitoB ratio in the liver with that in the muscle (Pearson’s *r* = 0.065, *P* = 0.787). Importantly, such differences in the MitoP/MitoB ratio within the muscle aliquots of an individual cannot be triggered by variation in the extraction and quantification of the compounds. Variation among samples in the efficiency of the extraction step would have an equal effect on both MitoB and MitoP, so would only change their absolute concentrations but not the MitoP/MitoB ratio. In a separate experiment, we showed that the quantification of each of the compounds and their isotopic spikes was very consistent, so that the very high repeatability in the calculated MitoP/MitoB ratio from duplicated quantification of individual extracts (ICC *r* = 0.878, n = 40, *P* < 0.001) is unlikely to be a source of variation in the estimation of the MitoP/MitoB ratio (see Appendix S3 in SI).

### Step 3: Determination of the effect of exposure duration and initial concentration of MitoB on MitoP/MitoB ratios

The body mass of free-ranging animals may be difficult to standardize and could give different initial concentrations of MitoB in the mitochondria of different fish injected with the same dose. A difference in the rate of reaction between MitoB and H_2_O_2_ could then affect MitoP/MitoB. On the other hand the MitoP/MitoB ratio would be expected to increase with length of exposure, and the timing of recapture of free-ranging subjects may be difficult to control. Furthermore, the compounds excretion, whose rates depend on concentration of compounds and exposure duration, might affect the ratio. In order to test for possible effects of these factors on the MitoP/MitoB ratios, 80 fish were injected with a standard dose of 50 nmol per fish. As the size of the fish ranged from 5.05 to 21.31 g (12.39 ± 0.43 g) but all fish received the same absolute dose of MitoB, the initial concentration of MitoB in the body (nmol MitoB g^−1^ fish) varied four–fold among individuals (Fig. 2A). Variation among individuals in the exposure durations of MitoB was caused by sampling liver from injected fish at slightly different post-injection times (range: 27–29 h). We used a general linear mixed model to test whether the measured MitoB or MitoP content in the sampled tissue was affected by the initial concentration of MitoB in the injected fish, the exact duration of exposure and the mass of sampled tissue, with processing extraction batch being included as a random effect.

Final MitoP and MitoB content in these liver samples varied up to 188-fold (range: 2.41 to 163.46 pmol) and 24-fold (range: 74.82 to 1782.02 pmol) respectively (n = 76, due to 4 fish having MitoP AA measurements below the reliable detection limit of the HPLC-MS). The size of the liver samples used for the extractions (range 8.30 to 56.20 mg, mean = 25.36 ± 1.39 mg) had no effect on the measured MitoP (F_1, 66.713_ = 0.161, *P* = 0.689) or MitoB content (F_1, 67.188_ = 0.822, *P* = 0.378). However, the duration of exposure to the MitoB probe (26.88 to 29.42 h, mean = 27.96 ± 0.07 h) had a small but significant negative effect on final MitoB content (F_1, 68.548_ = 7.689, *P* = 0.007, Fig. 2A) but no significant effect on MitoP content (F_1, 67.884_ = 1.055, *P* = 0.308). Finally, final MitoB content (i.e. about 28 h after injection) was slightly higher in smaller fish, i.e. those that started with a higher concentration of MitoB (F_1, 66.454_ = 5.406, *P* = 0.023, Fig. 2B). However, variation in initial MitoB concentration had no significant effect on final MitoP content (F_1, 66.105_ = 1.145, *P* = 0.289). These results suggested that it might be prudent to take initial whole-body MitoB concentration and exact duration of exposure into account when calculating MitoB content of the sampled tissue (whereas in our study MitoP levels did not require correction). In order to correct the MitoP/MitoB ratio for these effects, the MitoB content could be first standardized for the mean values of the exposure duration and the initial concentration of MitoB in the body. However, such corrections were found to have relatively little effect on calculated MitoP/MitoB ratios, since the ratios calculated from uncorrected and corrected MitoB content were very similar (ICC *r* = 0.939, n = 76, *P* < 0.001; Fig. 2C). For the rest of this study, MitoP/MitoB ratios were therefore calculated without applying any correction.

### Step 4: Applicability to aquatic models

In order to test whether MitoB and its derivate MitoP can transfer between fish and water, and so potentially between fish living in the same water body, we first injected isolated fish and then 24 h later sampled and flash-froze both the fish liver and the water of the tank in which each fish had been held (n = 3 fish held in separate tanks; see Appendix S4 in SI for protocol of probe extraction from water samples). The analyses showed that measurable levels of both MitoB and MitoP were detected in the livers of injected fish, but none were detected in the water in which they had been living (data not shown). In a separate trial, MitoB was added directly to the water of a 2 L fish tank containing a single non-injected fish; the three tanks in the experiment received different doses of MitoB (3, 30 or 300 μL of a 44.43 mM solution of MitoB in 100% ethanol), producing final MitoB concentrations in the water of 66 nM, 660 nM or 6.6 μM, respectively. When sampled 24 h later, MitoB was detectable in water at the two highest concentrations, but was undetectable in fish living in water at any of the 3 concentrations of MitoB (data not shown). Finally, to test for potential contamination of the probe between fish, a non-injected fish was put in the same tank as an injected fish (n = 4 pairs). Analysis of livers from non-injected fish (living for 24 h in the same tanks as injected fish) show that while typical levels of MitoB and MitoP were found in injected fish, none were detectable in the non-injected fish (data not shown). These trials indicate that the compounds do not transfer between fish in these experimental conditions, so allowing independent measurements to be made from fish living in the same water.

## Discussion

We have developed a step-by-step procedure (Steps 1–3) that is required in order to apply the MitoB method to a new animal model, with an additional step that is necessary before using the MitoB method on aquatic organisms (Step 4). In juvenile brown trout kept at 12 °C, we found that MitoB had already accumulated in the tissues 3 h post-injection, but levels of MitoP were in some cases too low to be accurately quantified. Larger tissue samples or a more sensitive HPLC-MS may overcome this detection limit and allow more reliable measurement of MitoP/MitoB ratios soon after MitoB injection. More importantly, MitoB was still present at high levels in the fish 72 h after injection, so allowing measurements of H_2_O_2_ levels in these experimental conditions over at least a 3 day period. However, validation work is still necessary to confirm the consistency of the time-normalized MitoP/MitoB ratio over a period of several days. In the meantime, we recommend using a duration of MitoB exposure that does not affect the time-normalized MitoP/MitoB ratio (see step 3). However, our results clearly show that the method can be used even if animals are not recaptured until several days after injection, so making it feasible to use the assay in the field. Note that this time course is likely to be temperature-dependent: MitoB was found to be barely detectable 12 h after injection in *Drosophila* living at 25 °C^19^, presumably due to high rates of excretion, so potential exposure durations are likely to be longest in temperate ectotherms and shortest in endotherms. The effect of body temperature on the time period over which the compounds remain measurable clearly needs to be considered in all new study systems.

After the selection of an appropriate experimental timescale (about 24 h in our case), the effects of (i) the dose of MitoB injected, and (ii) the exact duration of MitoB exposure need to be evaluated. We chose to inject a standardized dose of MitoB (constant volume and amount) instead of a body mass-normalized dose (constant amount per unit of body mass) since this represents the situation faced by many ecologists where measuring the body weight of mobile animals and adjusting the dose accordingly is not always convenient (e.g. when working in the field). In addition, the duration of MitoB exposure will depend on the recapture time of the animal, which can be variable when sampling free-living organisms. Our results show that the MitoB content at the end of the exposure was influenced by exposure duration and concentration of MitoB at injection; while these had a minimal effect on the variation in MitoP/MitoB ratio found in our data, this may not be the case in other contexts and so both should be quantified and their importance evaluated. Although the MitoB method necessities the capture, injection, release and then recapture of the animal, this is a very commonly used protocol for a number of well-established assays that assess physiological processes in an ecological context (e.g. the doubled labelled water method of measuring energy expenditure and the phytohaemagglutinin test for measuring acquired T-cell immunity^24^,^25^).

Several recent articles have raised important issues regarding the tissue-specificity of oxidative damage and anti-oxidant defences to measure oxidative stress^11^,^26^,^27^. In our study, no correlation was found between MitoP/MitoB ratios in the liver and in the white muscle across individuals. MitoP/MitoB ratios were much higher in the white muscle compared with the liver. Since MitoP/MitoB is a ratio, it provides a measure of the mean levels of mitochondrial H_2_O_2_ of the tissue regardless of the mitochondrial density. A higher MitoP/MitoB ratio in the muscle can be associated with a higher H_2_O_2_ levels per mitochondrion. However, interpretations of differences in MitoP/MitoB ratio between tissues on H_2_O_2_ levels must be informed by knowledge of the mitochondrial density in each tissue. This is because the ROS level in a tissue will depend not only on the ROS produced in the mitochondria^28^,^29^, but also the number of mitochondria and the capacity of antioxidant defences^30^,^31^,^32^. Differences in mitochondrial ROS levels between tissues are perhaps not surprising since the organs of an animal perform different functions and are potentially coping with very different H_2_O_2_ levels^11^. Disparities in oxidative stress can also occur within a tissue^33^,^34^. Indeed, our expectation that the MitoP/MitoB ratio would be the same within subsamples of a tissue was only partly met, since MitoP/MitoB ratios were consistent between subsamples of liver but not muscle. Muscle tissues usually display heterogeneity in their mitochondrial types and distribution even within a muscle type^35^,^36^, likely leading to local differences in the functioning of the mitochondria and consequently their ROS production. This is also supported by the study of Kuznetsov *et al*.^37^ showing mitochondrial heterogeneity within single muscle fibres. This heterogeneity of the MitoP/MitoB ratio means that researchers need to decide on which tissues *and* on the number of replicates to measure in order to obtain a representative assessment of mitochondrial ROS levels in the animal, as for other oxidative markers^11^,^26^,^27^.

Once these factors have been taken into account, the MitoB method offers many advantages over other ROS assays. First, unlike other current methods it offers flexibility in the timing and location of the laboratory steps of the protocol (extraction and quantification), since the sample can be frozen and stored at −80 °C before analysis^21^. Second, comparison of the results across experimenters and laboratories is possible since the MitoP/MitoB ratio can correct for variation in the amount of MitoB initially injected and in the efficiency of extraction and quantification. Third, the method can be used on small animals or tissues with low mitochondrial density since it requires only a few mg of tissue^19^,^23^,^38^. Finally, the MitoB molecule belongs to a group of compounds that can be used as *in vivo* exomarkers at non-toxic concentrations^18^,^38^,^39^,^40^. So far MitoB has been successfully injected into *Drosophila*, mice and fish^19^,^23^, with injected-fish moving, feeding and behaving normally (personal observation).

In summary, this method provides the means to move towards direct assessment of ROS levels in living animals from the size of a fly upwards, across a range of ecological contexts (i.e. field as well as laboratory) and over timescales from hours to days, all of which are relevant criteria for ecologists and evolutionary biologists^27^. For instance, the MitoB approach could be used to investigate the supposed increase in ROS during reproduction^6^ or rapid growth^41^ or the occurrence of ROS-mediated sexual selection^3^. However, we need to be careful over its interpretation. Analysis of a tissue’s MitoP/MitoB ratio by itself is insufficient to make inferences about the level of oxidative stress in the whole organism, since it is only measuring the imbalance between generation and scavenging of H_2_O_2_ in the mitochondria. For instance, H_2_O_2_ levels as measured using MitoB increase with age in flies but do not explain variation in lifespan and fecundity^19^,^22^, presumably because these life-history traits are also dependent on differential energy allocation to repair mechanisms^2^. In addition, it should be remembered that H_2_O_2_ also functions as a signalling molecule controlling essential cellular processes^42^,^43^. For instance, H_2_O_2_ is involved in developmental control and immune responses^33^,^44^. Because of these opposite functions, the “dose-response effect” of H_2_O_2_ needs attention: when, where and by how much do H_2_O_2_ levels constrain the animal? ROS have been suggested to act as universal life-history constraints^1^,^2^. However, there is little direct evidence for how ROS actually influence animal life-histories. It is currently unknown whether any ROS-induced constraints on animal performance are more likely to act through an accumulation of oxidative damage or through the diversion of energy towards ROS scavenging and repair systems and hence away from other body functions. The MitoB method provides one tool to address these questions, and so provides the information to evaluate the role of oxidative stress in life-history evolution.

## General Methods

### Ethics statement

This study adheres to the animal welfare standards of the U.K. Home Office. All animal experiments were conducted in accordance with the Guiding Principles for the Care and Use of Laboratory Animals. The study was covered by Home Office Project Licence No. 60/4292 and approved by the University of Glasgow Animal Welfare and Ethical Review Board.

### Preparation and conservation of the probe compounds

The probe compounds MitoB, MitoP, and their deuterium spikes deuterium_15_-MitoB (*d*_*15*_MitoB) and deuterium_15_-MitoP (*d*_*15*_MitoP) were synthesized at the University of Glasgow according to Cairns, *et al*.^45^ and kept as a crystalline solid at −20 °C. MitoB is already commercially available (Cayman Chemical, Ann Arbor, Michigan, USA), and commercial development of the other 3 compounds is ongoing, but in the meantime RCH can provide the four compounds to interested researchers on request. If large quantities are required, there is also a detailed published protocol for the synthesis of these compounds^45^. A MitoB solution was produced by dissolving MitoB powder in absolute ethanol heated to 60 °C; this was then diluted to 504 μM MitoB in a sterile saline solution (0.9% (w/v) NaCl/H_2_O), with a final ethanol content of 1% (v/v). A 504 μM MitoP solution was produced in the same way. Aliquots of MitoB, MitoP and 0.9% saline solutions were stored at −70 °C and thawed at the time of the experiment. Standard solutions for the calibration curve and for the deuterium spikes were made by serially diluting the compounds in 100% ethanol; these were also kept at −70 °C.

### Maintenance of animals

All the experiments were conducted on juvenile brown trout *Salmo trutta* at the University of Glasgow in 2014 and 2015. For each experiment, trout were initially held in a common tank under standard conditions of temperature (12 °C), photoperiod (12 L: 12 D), and *ad libitum* food (trout pellets, BioMar Ltd, Grangemouth, UK and Chironomid larvae, BCUK Aquatics Ltd, Lincolnshire, UK). At the start of each experiment, animals were transferred to individual tanks, unless specified, containing 2 L of aerated water, allowed to acclimate for 24 h before being injected with the probe, and maintained in these conditions throughout the period of probe exposure.

### Probe exposure

Fish were lightly anaesthetised (50 mg benzocaine per litre water) and then injected with MitoB into their intraperitoneal (IP) cavity using an insulin syringe (capacity 300 μl, 10 μl graduations). The injection was anterior to the base of the pelvic fins. Control animals were included in order to obtain a blank control value for the subsequent quantification analysis. Each fish was injected with the same volume (100 μl) of fluid, which was a standard dose of 504 μM MitoB (50 nmol/fish) or a saline solution (0.9% (weight in g/volume in mL) NaCl/H_2_O) in the case of controls. Insulin syringes have a very fine needle, but to avoid loss of any injected solution, a finger was kept on the punctured skin which was slowly massaged for a few seconds. The injected fish were put back in their well-aerated tanks which allowed a complete recovery according to Kennedy, *et al*.^46^ within less than 10 minutes. At the end of the period of exposure each fish was caught with a hand net and immediately culled by section of the spinal cord posterior to the skull. The tissues of interest (liver, but also muscle in one experiment) were rapidly dissected and flash-frozen in liquid nitrogen and stored at −70 °C until analysed. The time from capture to freezing of the sample was minimised (less than 3 min) to avoid effects of handling stress and cessation of blood circulation on mitochondrial properties. When specified, samples of water from the tanks in which the fish were held were also flash-frozen.

### Quantification of the compounds

**Figure 1:**
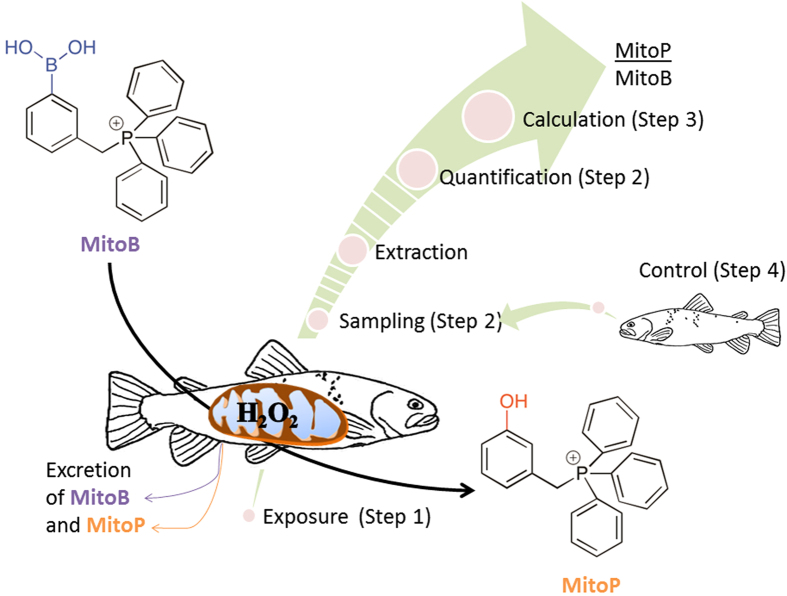
Schematic representation of the MitoB method. The animal is injected with the MitoB molecule. This becomes concentrated in the mitochondria, where it is converted to a stable alternative (MitoP) when reacting with hydrogen peroxide (H_2_O_2_), one major reactive oxygen species produced by the mitochondria. After an appropriate period of exposure (i.e. before the MitoB has all been excreted but after detectable amounts of MitoP have accumulated), samples are taken from the tissue(s) of interest; both MitoB and MitoP are then extracted, after having spiked the samples with known amounts of deuterium MitoB and deuterium MitoP (to determine extraction and quantification efficiency). The concentrations of MitoB, MitoP and their deuterated equivalents are determined by HPLC-MS, and the MitoP/MitoB ratio (the indicator of H_2_O_2_ levels) is calculated after taking account of any necessary correction factors. The dashed sections of the arrow represent the stages at which the protocol can be halted if samples are kept frozen. The diagram also indicates how each experiment described in the article address the assumptions of a particular step of the protocol; Step 4 tests whether there is any cross-contamination of MitoB or MitoP among individuals from the same water body (so validating its use in non-isolated aquatic animals).

**Figure 2:**
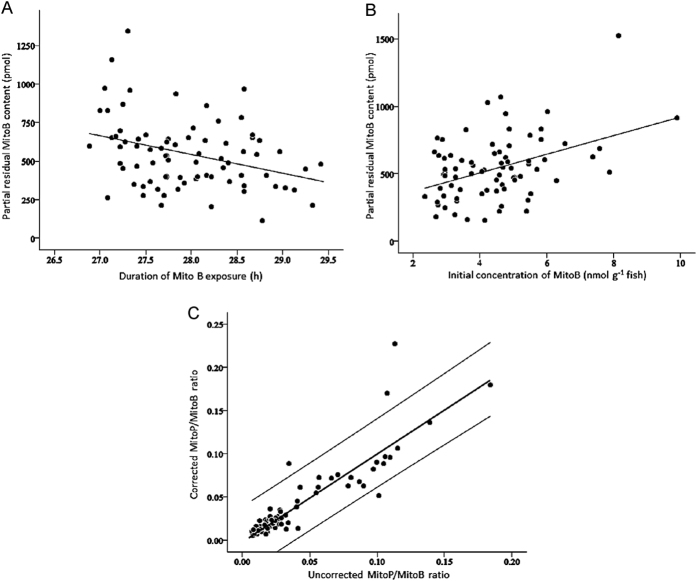
Effect on the measured MitoB content of (**A**) the exact duration of exposure to MitoB and (**B**) the concentration of MitoB at injection; both analyses based on liver samples of 76 fish. (**C**) The relationship in the same 76 samples between the MitoP/MitoB ratio calculated from uncorrected MitoB values and the ratio calculated from MitoB values corrected by standardization for the exposure duration and the concentration of MitoB at injection; the central thick line is the linear regression line and the two external thin lines represent the 95% confidence interval of the data.

**Figure 3:**
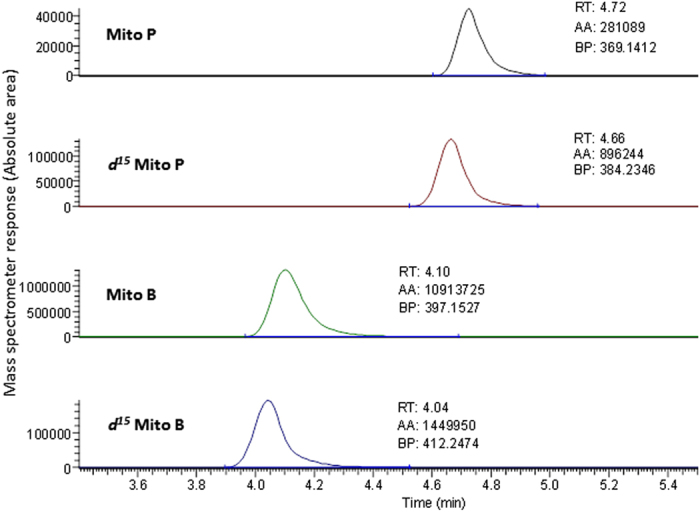
Example of chromatograms from the HPLC-MS analysis of Mito compounds within tissue samples of brown trout. MitoP, *d*_*15*_MitoP, MitoB and *d*_*15*_MitoB were measured simultaneously by HPLC-MS. Each chromatogram characterised a compound by its retention time (RT), its absolute area (AA) and the base peak (BP) that displays the molecular mass of the molecule of interest.

To measure the MitoP/MitoB ratio, the tissue is spiked with MitoB and MitoP isotopes, extracted, and then quantified by HPLC-MS (Fig. 1). Methods for extraction, quantification and analysis of the probe were adapted from Cochemé, *et al*.^21^ and are only briefly described here since more details can be found in Salin, *et al*.^23^. The standard operating procedure (SOP) documents important points to consider when developing the protocol on a different HPLC-MS or using new species or tissues (see Appendix S1 in SI). Calibration curves were generated using standards of MitoB, d15MitoB, MitoP and d15MitoP, prepared by serial 1:5 and 1:2 dilutions of stock solutions for each of the four compounds in ethanol which were then added to a solution containing 20% ACN, 0.1% FA and stored at −70 °C until analysed (Table S1a). Different standards were initially used to generate the calibration curves in Cochemé, *et al*.^21^. We compared both types of calibration curves to further validate standards used in our study (see Appendix S2 in Supplementary Information, SI). High performance liquid chromatography (HPLC) was used to separate MitoB and MitoP (along with their isotopic spikes); the relative concentrations of the four compounds were then quantified by mass-spectrometry (MS). Vials containing samples and standards were defrosted, vortexed for a few seconds, and placed in an auto sampler in a refrigerated holder at 4 °C until the end of the HPLC-MS analysis. Fish samples or standards were injected in the mobile phase of the Thermo Scientific Accela™ HPLC (Thermo Fisher Scientific, San Jose, CA, USA), and then directed to an Exactive Orbitrap trap MS (Thermo Fisher Scientific, Hemel, UK) as described in Salin, *et al*.^23^. For all analysed samples (control fish, treated fish and standards), the absolute area (AA) from the HPLC-MS output under each of the 4 peaks (i.e. for MitoP, *d*_*15*_MitoP, MitoB and *d*_*15*_MitoB) was analysed by Xcalibur software (Thermo Fisher Scientific, Fig. 3).

### Calculation of the MitoP/MitoB ratio

Peaks from the standards were used to produce calibration curves relating AA for each of the four compounds to pmol of MitoP, *d*_*15*_MitoP, MitoB and *d*_*15*_MitoB. The homogenates initially contained 100 pmol *d*_*15*_MitoB and 50 pmol *d*_*15*_MitoP as internal spikes, which allowed calculation of individual coefficients for the extraction and quantification efficiency of *d*_*15*_MitoB and *d*_*15*_MitoP. The values for MitoP and MitoB content (pmol) per sample were corrected using these individual coefficients. The HPLC-MS response for “blank” samples, i.e. those which have been exposed to saline solution with no MitoB, must be measured for each new animal model or tissue. In our study the AA of MitoP and MitoB for the control fish injected with saline solution were below the detection level, and so were not considered for calculation. For all other fish we calculated the ratio pmolMitoP/pmolMitoB (dimensionless, and corrected for extraction and quantification efficiency), hereafter referred to as the “MitoP/MitoB ratio”; higher values indicate a higher content of H_2_O_2_ in the mitochondria of the living animal^21^.

## Additional Information

**How to cite this article**: Salin, K. *et al*. Using the MitoB method to assess levels of reactive oxygen species in ecological studies of oxidative stress. *Sci. Rep.* **7**, 41228; doi: 10.1038/srep41228 (2017).

**Publisher's note:** Springer Nature remains neutral with regard to jurisdictional claims in published maps and institutional affiliations.

## Supplementary Material

Supplementary Information
