# Supplementary material for: Using the MitoB method to assess levels of reactive oxygen species in ecological studies of oxidative stress
Source: Sci Rep. 2017 Jan 24;7:41228. doi: 10.1038/srep41228 (PMC5259740; doi:10.1038/srep41228)
Supplement: Supplementary Information [file srep41228-s1.doc]

Supplemental information

**Using the MitoB method to assess levels of reactive oxygen species in ecological studies of oxidative stress**

*Karine Salin^1*^, Sonya K. Auer^1^, Eugenia M. Villasevil^1^, Graeme J. Anderson^1^, Andrew G. Cairns^2.^, William Mullen^3^, Richard C. Hartley^2^ and Neil B. Metcalfe^1^*

^1^ Institute of Biodiversity, Animal Health & Comparative Medicine, University of Glasgow, UK.

^2^ School of Chemistry, University of Glasgow, UK.

^3^ Institute of Cardiovascular and Medical Sciences, University of Glasgow, UK

*Author for correspondence: [salin.karine@gmail.com](mailto:salin.karine@gmail.com)

Key words: aquatic animal, HPLC-MS, hydrogen peroxide, ROS, tissue-specificity, wild population.

Appendix S1: STANDARD OPERATING PROCEDURE FOR HPLC-MS TUNING

All quantification of the compounds MitoP, *d_15_*MitoP, MitoB and *d_15_*MitoB, using a high performance liquid chromatography system connected to a mass spectrometer (HPLC-MS), were conducted following standard operating procedures based on the following guidelines. We present how the method was conducted in our study as an example (but non-exclusive way) and then we present solutions to frequently encountered issues.

*Instrumentation and chemical*

To separate and introduce the compounds into the MS a liquid chromatography system is required, while quantification of the compound in the eluant coming from the HPLC requires a mass spectrometer. This can either be a HPLC system or an ultra-performance liquid chromatography (UPLC) system using a 2.0 mm column running at 200 µl/min or a 4.6 mm column running at 1 ml/min. The chromatographic column used in the present study is a standard reverse phase C 18 column such as a hypersil gold column (150 mm x 2 mm, I.D. 2.0μm) with guard column. Standards at known concentration of isolated compounds are necessary to set up the quantification of the 4 compounds. The concentration of the standards used to provide calibration curve is highly dependent on the system used. Table S1a gives a suggested range of starting concentrations for a conventional HPLC 4.6 mm column (flowrate 1 ml/min) and UPLC 2.0 mm column (flowrate 200 µl/min).

*Mass spectrometric detection of the 4 compounds*

Two types of mass analysers have been used to detect Mito compounds in biological samples: one based on high resolution accurate mass (HRAM) ^1^, and the other on tandem mass analysis (MS/MS) ^2^. Mass spectrometric analysis was carried out using positive ion mode. To tune the HRAM or the MS/MS, directly infuse the isolated compound into the MS using an appropriately diluted stock solution containing only a single compound, starting at a mid-range concentration to visualize any low level contaminants while not overloading the mass spectrometer with the compound of interest (e.g. Standard 4 in Table S1a). Detect the compound on the ion spectrum according to the accurate mass of the ions or the approximate one for the HRAM and MS/MS, respectively (Table S2a and b). This provides evidence of the purity of each standard and allows tuning of the mass spectrometer on each compound if needed. In the case of MS/MS analysis this procedure was used to optimise the fragmentation energy. The fragmentation energy used in our experiments carried out in an ion trap mass spectrometer (Thermo Fisher Scientific) was 55%.

*Coupling of the chromatographic system to the mass spectrometer*

First, the stock solution should be checked for impurities by HPLC-MS analysis at high concentration. Second, a mixture of the 4 standards is assessed to confirm separation of the peaks of MitoB compounds from the MitoP compounds. In the setup described here there was approximately one minute separation between the pairs of peaks P *versus* B. Finally, an extended calibration curve is performed to ensure the linear range of the system and the minimum and maximum concentrations that can be quantified for each of the 4 compounds. Note that molecules that differ only in the degree of deuteration can show small differences in their interactions with the stationary phase during separation by HPLC, so that the deuterium compounds elute slightly more quickly than their isotope ^3-5^ – this is evident in figure 2 of the main article. For this reason, it is important to integrate the amount of the deuterated and undeuterated compounds over the whole peak area in place of peak height.

*Sequence of the gradient in the mobile phase*

The mobile phase used in our analysis was based on solvent A: 98% water 2% acetonitrile in 0.1% formic acid and solvent B 100% acetonitrile with 0.1% formic acid. The gradient that allowed separation of the four MitoP, *d_15_*MitoP, MitoB and *d_15_*MitoB compounds was based on a starting solvent of 30% B, rising to 65% solvent B over 10 minutes prior to a wash and re-equilibration phase.

*Integration of the peak area*

The data was quantified by integrating the area under each peak. In the software package used in our experiments (Xcalibur version 2.0.7) the significant parameters used were:

Integration algorithm: Genesis

Percentage of highest peak: 1%

Minimum peak height (S/N): 2

S/N threshold: 0.5

***Possible issues:***

- If the pairs of compounds P and B overlap, the proportion of solvent B in the elution should be decreased to allow slower migration of the probes, which should improve separation without adversely affecting the peak width of the probes.
- If the concentration of the probe is too high this may result in a carry-over effect (causing cross-contamination between runs, as part of the sample is not completely washed off the column from the previous injection).
- If a peak in a sample is below the limit of quantification of the calibration curves, then its quantification cannot be made. A possible solution is to increase the volume of injection, but it is necessary to ensure that the volume injected does not overload the column for the other compounds causing asymmetric or flattened peaks.
- If only MitoP is below the linear section of the calibration curves, it may mean that the exposure duration was not long enough to display sufficient accumulation of MitoP in the tissue of interest and the size of the sample extracted. This can be overcome by increasing the exposure time duration and / or the amount of extracted sample.
- If no probe peaks are detected after injection of a sample while the compounds in the standards are seen, the issue may come from the sample itself. This can be because they contain high levels of compounds that can cause suppression of ionisation of the compounds of interest, usually during or immediately after elution of the void volume peak (Fig. S1). The elution gradient should allow sufficient time for the ion current to re-stabilise before the probes elute into the mass spectrometer.
- Another ion suppression type problem can, and did occur, due to a very high level of an unknown compound in samples that suppressed detection of closely eluting probes ^6^. We were unable to resolve this issue using chromatographic separation. If analysis is by full scan HRAM a possible solution is to split the scanned mass range so that this ion is not included for mass analysis. Alternatively, a selected ion monitoring approach can also work to eliminate this type of problem.

Appendix S2: COMPARISON OF STANDARDS TO BUILD THE CALIBRATION CURVE

In our study, calibration curves were generated using standards prepared by serial dilutions of stock solutions of the four compounds, without any tissue sample, as in Salin, et al^. 1^. Cochemé, et al^. 2^ in the original protocol prepared the standards for the calibration curve using a different approach: the standards were processed with a tissue sample and various amounts of MitoB and MitoP but constant amounts of deuterium compounds. We compared both types of calibration curves, hereafter called *calibration 1* and *calibration 2* for standards prepared according to Cochemé, et al^. 2^ and our study, respectively. For *calibration 1*, standards were prepared by serial 1:5 and 1:2 dilutions of a MitoB and MitoP stock solution (Table S1b) and were added to control samples of liver tissue from unexposed fish. Tissues were then homogenised with the MitoB standard, the MitoP standard and the spike. Standards were processed by following the same extraction protocol as was used for the experimental liver samples. For *calibration 2*, calibration curves were generated using standards of MitoB, *d_15_*MitoB, MitoP and *d_15_*MitoP, prepared by serial 1:5 and 1:2 dilutions of stock solutions of each of the four compounds in ethanol which were then directly added to a solution containing 20% ACN, 0.1 % FA (table S1a). To test the consistency of the MitoP/MitoB ratios calculated from *calibration 1* and *2*, the quantification of probe levels in liver samples from 40 individuals exposed to MitoB for 24h were run in a single reading set along with standards for both *calibrations 1* and *2*. For *calibration 2*, calculation of the MitoP/MitoB ratio was done as described in the section “calculation of the MitoP/MitoB ratio” in the main text. For *calibration 1*, calculation of the MitoP/MitoB ratio differed. The ratios of AA MitoB/AA *d_15_*MitoB and AA MitoP/AA *d_15_*MitoP were calculated for the standards and the samples. The calibration curves of AA MitoB /AA *d_15_*MitoB and AA MitoP/AA *d_15_*MitoP against pmol of MitoB and MitoP was then generated. The MitoB and MitoP content for each liver samples from the 40 individuals exposed to MitoB was calculated by converting the AA MitoB/AA *d_15_*MitoB and AA MitoP/ AA *d_15_*MitoP ratios into pmol using the appropriate calibration curve. The calculated values for the MitoP/MitoB ratio were slightly smaller when using *calibration 2* (Mean ± SE: 0.0731 ± 0.0063) than when using *calibration 1* (Mean ± SE: 0.0877 ± 0.0076). However, the level of consistency of the MitoP/MitoB ratio between the values calculated from the two calibration curves was very high (Fig. S2, ICC r = 0.959, p < 0.001). For the rest of the study, MitoP/MitoB ratios were calculated using *calibration 2* since it allows as accurate a calculation as *calibration 1* while determining of standard curve and detection limits for each compounds, saving the time of the extraction step and removing the need to sacrifice additional animals for control tissues.

Appendix S3: REPEATABILITY OF THE QUANTIFICATION OF THE COMPOUNDS AND MitoP/MitoB RATIOS

To assess the HPLC-MS repeatability, the quantification of probe levels in extracts of liver samples from 40 individuals exposed to MitoB for 24 h were run in duplicate in a single reading set, along with duplication of the calibration curve. Intra class correlation coefficients (ICC) were used to test for the consistency of the quantification of the compounds between the two measures from the same tissue extract. The resulting repeatability was high for the absolute area of the four compounds (MitoP: ICC *r* = 0.983, *P* < 0.001, *d_15_*MitoP: ICC *r* = 0.703, *P* < 0.001, MitoB: ICC *r* = 0.974, *P* < 0.001, *d_15_*MitoB: ICC *r* = 0.598, *P* < 0.001; in all cases n = 40; Fig. S3A to S3D). The resulting repeatability in the calculated MitoP/MitoB ratios was also very high (ICC *r* = 0.878, n = 40, *P* < 0.001; Fig. S3E). Note that the MitoP content was below the detection limit of the HPLC-MS in two samples (outliers 1 and 2). In both samples, the AA of MitoP was below the range of linearity of the standard curve; this was not due to a detection failure since the measurements of the MitoP deuterium spike in the samples were normal. Instead it is likely that too little probe was extracted, either because the tissue sample was too small (the liver sample from individual 1 was the smallest of those analysed) or the homogenization process was inadequate.

Appendix S4: EXTRACTION OF THE MITOB AND MITOP FROM THE WATER.

To extract MitoB and MitoP from water, each water sample (780 µL) was added to acetonitrile (ACN) and formic acid (FA) in order to obtain a final solution containing 60% (v/v) ACN and 0.1 % (v/v) FA. After centrifuging for 10 min at 16,000 g, the supernatant was centrifuged for a final 10 min at 4,560g in microcentrifuge filters. The filtered solution from the water samples was processed as done for the tissue samples from the drying step onwards ^1^.

**References**

Table S1: Amount of the probes in the standards used to generate calibration curves of (a) MitoP, *d_15_*MitoP, MitoB and *d_15_*MitoB, and (b) of MitoP/*d_15_*MitoP and MitoB/*d_15_*MitoB.

a.

| **Standard** | **MitoP (pmol)** | ***d_15_*MitoP (pmol)** | **MitoB (pmol)** | ***d_15_*MitoB (pmol)** |
| --- | --- | --- | --- | --- |
| **1** | 1313 | 100 | 5040 | 200 |
| **2** | 656.5 | 50 | 2520 | 100 |
| **3** | 131.3 | 10 | 504 | 20 |
| **4** | 65.65 | 5 | 252 | 10 |
| **5** | 13.13 | 1 | 50.4 | 2 |
| **6** | 6.565 | 0.5 | 25.2 | 1 |
| **7** | 1.313 | 0.1 | 5.04 | 0.2 |
| **8** | 0.6565 | 0.05 | 2.52 | 0.1 |
| **9** | 0.32825 | 0.025 | 1.26 | 0.05 |
| **10** | 0 | 0 | 0 | 0 |

b.

| **Standard** | **MitoB** **(pmol)** | **MitoP** **(pmol)** | ***d_15_*MitoB** **(pmol)** | ***d_15_*MitoP** **(pmol)** |
| --- | --- | --- | --- | --- |
| **1** | 2520 | 656.50 | 100 | 50 |
| **2** | 504 | 131.30 | 100 | 50 |
| **3** | 252 | 65.65 | 100 | 50 |
| **4** | 50.40 | 13.13 | 100 | 50 |
| **5** | 25.20 | 6.57 | 100 | 50 |
| **6** | 5.04 | 1.31 | 100 | 50 |
| **7** | 2.52 | 0.66 | 100 | 50 |
| **8** | 0 | 0 | 100 | 50 |

Table S2: Mass of the four ions detected by (a) the high resolution accurate mass and (b) the tandem mass analysis.

a.

| **Compounds** | **Accurate mass M+1** |
| --- | --- |
| **MitoP** | 369.141 |
| ***d_15_*MitoP** | 384.234 |
| **MitoB** | 397.153 |
| ***d_15_*MitoB** | 412.247 |

b.

| **Compounds** | **Approximate mass M+1** | **Mass daughter ions** |
| --- | --- | --- |
| **MitoP** | 369.2 | 183-185 and 260-263 |
| ***d_15_*MitoP** | 384.2 | 183-185 and 260-263 |
| **MitoB** | 397.2 | 191-195 and 275-279 |
| ***d_15_*MitoB** | 412.3 | 191-195 and 275-279 |

Table S3: Cross sectional assay of MitoB and MitoP content in the liver of brown trout exposed to one of the compounds for different durations (n=4 per time point and per compound). Note that the complete disappearence of MitoB and MitoP can be expected over time, however, a much longer time course would be necessary to observe this phenomenon in this system.

| **Injected compound** | **Exposure duration**  **± SE (hour)** | **Final concentration of compound**  **± SE (pMol/mg liver)** | **Generated MitoP ± SE; min-max (pMol/mg liver)** |
| --- | --- | --- | --- |
| **MitoB** | 3.3 ± 0.1 | 68.03 ± 18.56 | 4.63 ± 3.55 (0.00-15.16) |
| **MitoB** | 11.9 ± 0.1 | 84.21 ± 18.62 | 10.05 ± 6.65 (0.41-22.30) |
| **MitoB** | 24.2 ± 0.0 | 75.40 ± 14.06 | 2.16 ± 1.20 (0.00-5.59) |
| **MitoB** | 48.1 ± 0.0 | 62.42 ± 8.49 | 2.14 ± 0.44 (0.92-3.01) |
| **MitoB** | 72.0 ± 0.0 | 63.70 ± 26.28 | 2.66 ± 0.82 (0.86-4.34) |
| **MitoP** | 3.3 ± 0.1 | 93.77 ± 39.04 |  |
| **MitoP** | 11.9 ± 0.1 | 44.72 ± 14.24 |  |
| **MitoP** | 24.3 ± 0.0 | 127.16 ± 49.79 |  |
| **MitoP** | 48.2 ± 0.0 | 72.91 ± 11.45 |  |
| **MitoP** | 72.0 ± 0.0 | 76.92 ± 19.24 |  |

Fig. S1: Example of chromatograms from the HPLC-MS analysis of Mito compounds within samples of trout muscle with ion suppression phenomenon, i.e. a loss of signal between min 1 and min 2.5, just before the peak of interest. The same phenomenon could occur just at the same time as the peak immersion, so it would be necessary to split the spectrum of analysis into two spectra excluding the contaminant ion.

**
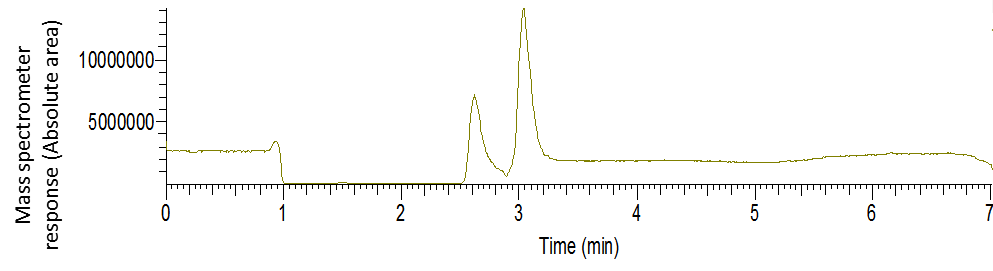
**

Fig. S2: Consistency of the MitoP/MitoB ratio as calculated using two different calibration approaches: calculated from standard curves for 4 compounds in the absence of tissue samples (*calibration 2*, y axis as in Salin, et al^. 1^) or from standard curves based on the compounds MitoB and MitoP together with a fixed amount of each spike and with a tissue sample (*calibration 1*, x axis as in Cochemé, et al^. 2^). **The central line is the linear regression line and the two external lines represent the 95% confidence interval of the data.**

Fig. S3: The repeatability of the quantification of probe levels by HPLC-MS was tested by running duplicate extracts of liver samples from 40 individuals exposed to MitoB for about 24 h. The replicability of HPLC-MS measurements was very high, as assessed by the relationships between two **quantifications** of (A**) MitoP, (B) *d_15_*MitoP, (C) MitoB and (D) *d_15_*MitoB** absolute areas (AA) **by HPLC-MS.** The values for **MitoP and Mito B content (pmol) per sample were calculated from the calibration curves and then corrected using the sample’s own coefficients for extraction efficiency calculated from the *d_15_*MitoP and *d_15_*Mito B content. (E) The r**eplicability of HPLC-MS measurements was confirmed by the strong similarity of the **MitoP/MitoB ratios** ca**lculated for the two samples taken from the same extract. The central thick line is the linear regression line and the two external thin lines represent the 95% confidence interval of the data; data points labelled 1and 2 refer to potential outliers discussed in the text. Insect of the graph A. shows the data points near the origins.**

A.

B.

C.

D.

E.
